# Zebrafish as a model to assess cancer heterogeneity, progression and relapse

**DOI:** 10.1242/dmm.015842

**Published:** 2014-07

**Authors:** Jessica S. Blackburn, David M. Langenau

**Affiliations:** 1 Department of Molecular Pathology, Regenerative Medicine and Center for Cancer Research, Massachusetts General Hospital, Charlestown, MA 02129, USA.; 2 Harvard Stem Cell Institute, Cambridge, MA 02139, USA.

**Keywords:** Cancer stem cell, Fluorescence, Intratumoral, Single cell, Targeted therapy, Tumor

## Abstract

Clonal evolution is the process by which genetic and epigenetic diversity is created within malignant tumor cells. This process culminates in a heterogeneous tumor, consisting of multiple subpopulations of cancer cells that often do not contain the same underlying mutations. Continuous selective pressure permits outgrowth of clones that harbor lesions that are capable of enhancing disease progression, including those that contribute to therapy resistance, metastasis and relapse. Clonal evolution and the resulting intratumoral heterogeneity pose a substantial challenge to biomarker identification, personalized cancer therapies and the discovery of underlying driver mutations in cancer. The purpose of this Review is to highlight the unique strengths of zebrafish cancer models in assessing the roles that intratumoral heterogeneity and clonal evolution play in cancer, including transgenesis, imaging technologies, high-throughput cell transplantation approaches and *in vivo* single-cell functional assays.

## Intratumoral heterogeneity and clonal evolution

**Fig. 1.**
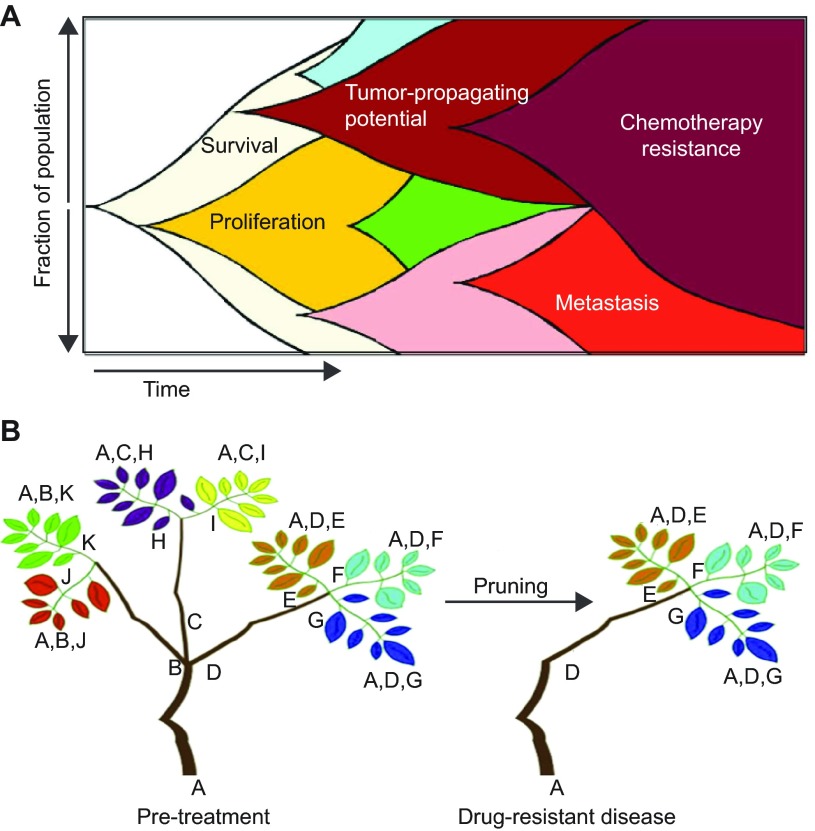
**Intratumoral heterogeneity and clonal evolution contribute to tumor progression, drug resistance and relapse.** (A) Schematic of clonal evolution in cancer, in which all cells in the tumor originate from a single cell. Genetically divergent clones develop over time that have different functional properties (some examples of which are labeled) and yet co-exist with other clones within the tumor simultaneously. Adapted, with permission, from Marusyk and Polyak (Marusyk and Polyak, 2010). (B) Schematic of a phylogenetic ‘tree’ generated through analysis of sequencing data. Several founding mutations can be identified in every clone (mutations A–D). Acquired mutations are generated over time, resulting in extensive genetic heterogeneity, which are represented by the branches and different colored leaves of the tree. Targeted therapy ‘prunes’ the branches, selecting for drug-resistance mutations. Adapted, with permission, from Murugaesu et al. (Murugaesu et al., 2013).

Cancer progression is a dynamic and stochastic process in which heritable genotypic and phenotypic changes are randomly produced as a result of increased genomic instability and/or epigenetic changes. Cells constantly undergo Darwinian selection, in which only those lesions that enhance fitness are propagated. This process is commonly referred to as clonal evolution (Swanton, 2012; Almendro et al., 2013). Ultimately, clonal evolution culminates in the production of a tumor comprising multiple, genetically distinct clones, each of which is represented at different cell frequencies within the bulk tumor. The clonal composition of a tumor will dynamically change, depending on the selective pressures present during various stages of tumor progression (Marusyk and Polyak, 2010). For example, early tumor development likely selects for mutations that afford proliferative or survival advantages, whereas later stages would select for those lesions that increase invasiveness, enhance tumor propagating potential, and/or provide resistance to metabolic stress or chemotherapy. Although some clones become evolutionary dead-ends, others are retained and can continue to undergo continued clonal evolution to acquire *de novo* mutations that alter their cellular phenotype (Fig. 1A).

Karyotypic analysis of chromosomes and fluorescence *in situ* hybridization (FISH) provided the first evidence that multiple, genetically distinct clones are present within a single tumor (Coons et al., 1995; Teixeira et al., 1996). More recently, next-generation sequencing has allowed for unbiased identification of mutations that are found within tumor cell clones, and has unveiled the startling extent to which genetic diversity and intratumoral heterogeneity are present within most human cancers. Navin and colleagues used comparative genomic hybridization techniques to demonstrate that multiple, genetically distinct clones were present within single breast cancer samples, and clonal subpopulations also varied between biopsies taken at different locations within the same tumor (Navin et al., 2010). Similar results have been observed in a wide-range of solid tumors and leukemia, suggesting that heterogeneity is a common trait among cancers (Götte et al., 2004; Campbell et al., 2010; Yachida et al., 2010; Anderson et al., 2011; Navin et al., 2011; Snuderl et al., 2011; Buob et al., 2012; Shah et al., 2012; Morrison et al., 2014). Next-generation sequencing and mathematical modeling have also provided unprecedented insight into the longitudinal sequence by which mutations are acquired as cancer cells clonally evolve (Fig. 1B). For example, analysis of acute lymphoblastic leukemia showed that the majority of relapse clones were related to those found at diagnosis but had also acquired new genetic lesions, the most common being biallelic loss of cyclin-dependent kinase inhibitor *CDKN2A/B* (Mullighan et al., 2008). In glioblastoma, clones that harbor mutations in the tumor suppressor gene tumor protein p53 (*TP53*) and *CDKN2A* underwent a branched evolution, with each clone ultimately differing in amplifications of receptor tyrosine kinases, such as *EGFR*, *PDGFRA* and *MET* (Snuderl et al., 2011). Although these types of phylogenetic studies document the order by which somatic mutations are acquired during tumor progression, the extent to which the lesions directly contribute to tumor onset, progression, relapse and metastasis often cannot be inferred.

## The clinical impact of intratumoral heterogeneity

The presence of clonal heterogeneity in cancer has profound clinical implications. Targeted therapies have recently emerged as a powerful tool to exploit a tumor’s dependence on critical survival pathways and have had good success against a variety of cancer types (Sosman et al., 2012; Cerrano et al., 2013; Swain et al., 2013). However, not all patients respond, and patients with advanced-stage cancers will eventually relapse. This might be attributed to the existence of inherently drug-resistant clones that are present even before treatment begins. For example, acute myelogenous leukemia, acute lymphoblastic leukemia and multiple myeloma commonly relapse from a rare, underrepresented clone contained within the primary leukemia; cells in this clone likely harbor mutations that impart therapy resistance (Mullighan et al., 2008; Ding et al., 2012; Keats et al., 2012). In chronic lymphocytic leukemia, clones with either splicing factor 3B1 (*SF3B1*) or *TP53* mutation found at diagnosis exhibited faster time to relapse irrespective of clone frequency, suggestive that these pre-existing mutations drive therapy responses (Landau et al., 2013). Similar results have been reported in a large number of solid tumors, suggesting that inherently resistant clones expand after treatment to drive relapse growth (Castellarin et al., 2013; Roche-Lestienne et al., 2002; Su et al., 2012). Importantly, therapy resistance can also evolve over time, through *de novo* mutations within a given clone (Heng et al., 2010; Murugaesu et al., 2013). Many cases of chronic myelogenous leukemia are caused by a translocation resulting in a fusion gene, *BCR-ABL*, which encodes a constitutively active tyrosine kinase. Treatment with the tyrosine kinase inhibitor imatinib is a common therapeutic approach for the disease. The majority of patients who relapse after initially showing a response to imatinib have acquired mutations in the *BCR-ABL* kinase domain that prevent imatinib binding, rendering these clones resistant to the drug (Shah et al., 2002). This type of evolution has been documented in other leukemias and solid tumors, suggesting that acquired resistance is a common feature of relapse (Diaz et al., 2012; Ding et al., 2012; Awad et al., 2013). Finally, cytotoxic chemotherapies often elevate mutation rates within cancer cells (Ding et al., 2012; Keats et al., 2012; Swanton, 2012), raising the possibility that drugs meant to help patients actually contribute to relapse by enhancing clonal evolution and expanding intratumoral heterogeneity.

Despite the clear clinical significance of intratumoral heterogeneity in predicting cancer progression, the subject remains poorly explored scientifically. The mechanisms of genomic instability that lead to clonal evolution and intratumoral heterogeneity are of crucial importance, yet are not well understood. Prospective studies are needed to identify the clonally dominant drivers of cancer progression, as well as to determine how a heterogeneous tumor responds to drug treatment and whether heterogeneity changes in response to therapy. To date, the majority of research that has focused on cancer heterogeneity has relied on mouse xenograft models and retrospective analyses of primary patient samples. Zebrafish cancers, which are histologically and genetically similar to human disease (White et al., 2013), have the potential to play a key role in our understanding of cancer heterogeneity, in large part owing to their fecundity, the low cost of husbandry and ease of transgenesis. For example, large-scale transgenesis utilizing hundreds of embryos per day, coupled with in-depth functional studies, can be used to cull the massive amount of bioinformatics data generated from patient samples to identify cancer drivers. The optical clarity of zebrafish larvae, combined with tumor cell labeling and imaging techniques, can resolve the effects of heterogeneity on cancer progression at the single-cell level and in real time. Single-cell tumor transplantation into thousands of recipient zebrafish, combined with *in vivo* chemotherapy treatments, can be used to functionally assess heterogeneity and follow clonal evolution in response to drug. Although no single model or analysis can accurately recapitulate the continuously evolving complexity of heterogeneous cancers, this Review will highlight how cancer biologists can take advantage of the unique strengths of zebrafish cancer models to assess the *in vivo* biological role of heterogeneity in cancer progression and patient outcome.

## Identifying common drivers of cancer progression through cross-species tumor comparisons

Application of bioinformatics algorithms to longitudinal array comparative genomic hybridization (aCGH) and genomic sequencing data has proven invaluable in tracing the mutational lineages of clones within human cancer. These data sets have provided a rich resource for identifying putative drivers of tumor progression. However, these experiments often identify hundreds of candidate oncogenes and tumor suppressors, and cannot differentiate between driver lesions that are required for tumor initiation, progression or continued growth and passenger mutations that impart no selective advantage to the cancer cell. Cross-species comparisons between tumor subtypes can narrow the list of potential drivers because genetic mutations that are common to cancers arising in diverse species are more likely to contribute to tumor progression. Over 70% of human protein-coding genes are related to zebrafish genes (Howe et al., 2013), yet, because zebrafish and human are evolutionarily distant, the syntenic blocks shared between the species are much smaller than those shared between human and mouse (Catchen et al., 2011; Zhang et al., 2013). Thus, focally amplified and deleted regions common to both human and zebrafish cancer share few co-segregating genes, making it easier to identify true oncogenic drivers. Among the first to apply high-resolution aCGH and cross-species comparisons to zebrafish cancer models, Zhang and colleagues showed that the aneuploid nature of human malignant peripheral nerve sheath tumors (MPNSTs) is recapitulated in zebrafish MPNST (Zhang et al., 2013). Eight genes were identified that occur as focal gains in both zebrafish and human cancers, one of which, fibroblast growth factor (*fgf6a*), accelerated MPNST onset in the zebrafish model, suggesting that it could also be an important driver in human disease (Zhang et al., 2010). aCGH analysis has also been utilized to identify commonly amplified genes across zebrafish and human rhabdomyosarcomas (RMSs), a pediatric cancer of the muscle (Chen et al., 2013). In this work, aCGH revealed 18 copy-number abnormalities (CNAs) common to both zebrafish and human RMS, most of which contained amplification of only one to three genes. Functional studies identified four genes that were required for continued tumor growth in human RMS cell lines, and thus might be good candidates for targeted therapy. Expression of the endothelial growth factor *VEGFA* was also significantly associated with poor prognosis in a subset of RMS patients, suggesting that the gene, which has not previously been associated with human RMS, is an important prognostic marker for the disease. Rudner and colleagues conducted iterative allotransplantation (transplantation to a genetically non-identical recipient of the same species) of zebrafish T-cell acute lymphoblastic leukemia (T-ALL) to select for aggressive disease, and observed that CNAs in serially passaged T-ALL encompassed oncogenes and tumor suppressor genes known to be crucial for human T-ALL progression, such as *notch*, *hes1* and *phf6* (Rudner et al., 2011). Cross-species comparisons also identified four novel CNAs that were associated with poor patient outcome (Rudner et al., 2011). In particular, the amplification of *C7ORF60*, a gene of unknown function, was associated with a <20% chance of event-free survival in T-ALL patients. Together, these studies provide proof-of-principle that the fundamental properties governing cancer progression have persisted throughout evolution, and that cross-species comparisons between heterogeneous human and zebrafish cancers can be used to identify clonally dominant driver mutations.

## Rapidly identifying oncogenic drivers using transgenic and mutant zebrafish lines

Large-scale genetic approaches can be readily used in zebrafish to rapidly assess whether putative drivers play a functional role in cancer initiation, progression and therapy resistance. The development of transposon-mediated transgenesis in zebrafish, using simple microinjection of RNA and DNA constructs into fertilized single-cell oocytes, results in 50–80% germline transgenesis (Balciunas and Ekker, 2005; Suster et al., 2009), a process that is far more efficient and cost-effective than the generation of transgenic mice. Mosaic transgenic approaches have also been recently utilized to rapidly identify collaborating oncogenes. In these studies, a candidate oncogene and a known tumor-inducing oncogene are placed under the control of a tissue-specific promoter and co-injected into single-cell oocytes. A proportion of the resulting mosaic transgenic zebrafish will integrate the DNA into the genome and co-express the transgenes in the correct target tissue, leading to the development of cancer (Langenau et al., 2008; Ceol et al., 2011; Blackburn et al., 2012). This system is ideal for large-scale screens that aim to identify collaborating oncogenes. For example, Ceol and colleagues utilized mosaic transgenic approaches to overexpress 18 individual genes in a zebrafish model of BRAF(V600E)-mutated *tp53*-deficient melanoma (Ceol et al., 2011). Genes from this screen were confined to the recurrent Ch1q21 amplification found in human melanoma (Ceol et al., 2011). From this analysis, the authors were able to identify the single driver oncogene found in this amplicon, the histone methyltransferase *SETDB1*, which significantly accelerated tumor onset. They went on to provide functional evidence that *SETDB1* changes histone methylation patterns to promote cancer progression, using both zebrafish and human melanomas. This screen required the generation of over 3000 transgenic animals; however, a timeframe of only 25 weeks was required to go from oocyte injection of genes of interest to melanoma formation. The scale and rapidity with which this transgenic study was conducted is simply not feasible in murine cancer models. Similar studies have been conducted in zebrafish to define the roles of potential cancer-causing genes in specific malignancies, including the importance of *ALK* in *MYCN*-induced neuroblastoma onset (Zhu et al., 2012), the contribution of *UHRF1* to genomic instability in hepatocellular carcinoma (Mudbhary et al., 2014), and the role of *BCL2* in intravasation and dissemination of T-ALL (Feng et al., 2010).

Genome-editing technologies have recently emerged as powerful tools for large-scale loss-of-function studies in zebrafish and will provide new ways to assess tumor suppressor function in zebrafish cancer. Genome-editing tools such as ZFNs (zinc-finger nucleases), TALENs (transcription activator-like effector nucleases) and CRISPR-Cas (clustered regularly interspaced short palindromic repeats/CRISPR-associated proteins) induce targeted mutations in genes through the generation of double-strand breaks and error-prone repair by non-homologous end joining (Sander et al., 2011; Moore et al., 2012). It was recently shown that a TALEN system known as GoldyTALEN can induce somatic lesions in zebrafish, which occurred with up to 86% efficiency in 14 of 14 GoldyTALENs tested (Bedell et al., 2012). Germline transmission in this system ranged from 18 to 100%. TALENs can also be used to generate heritable knock-in of large DNA fragments by homologous recombination with double-stranded donor DNA (Zu et al., 2013), facilitating gene replacement of knocked-out genes with fluorescent reporters or the creation of oncogenic point mutations within targeted genes. Unlike TALENs, which can be laborious to synthesize, the recently developed CRISPR/Cas system requires only single-step cloning of inexpensive oligonucleotides that are directed against the target DNA sequence. CRISPR/Cas has been shown to generate mosaic, biallelic and heritable mutations in zebrafish with 24–99% efficiency (Hwang et al., 2013; Jao et al., 2013), and can also facilitate knock-in alleles or introduce *loxP* sites (Chang et al., 2013; Hruscha et al., 2013). Unlike ZFNs and TALENs, the CRISPR/Cas system is capable of multiplex gene targeting (Jao et al., 2013), which would allow for targeting of multiple tumor suppressors in a cancer model. Although CRISPR/Cas is a powerful genome-editing tool, issues have recently arisen regarding specificity and frequent off-target mutations (Fu et al., 2013; Fu et al., 2014), which are less of a problem for ZNFs and TALENs. Work will undoubtedly continue to make these techniques more robust and reliable. Short-hairpin RNA (shRNA) technology has also recently been adapted to the zebrafish to knock down expression of genes of interest *in vivo* (De Rienzo et al., 2012), and hairpins have been generated that are heritable and can be spatiotemporally controlled using the *Gal4/UAS* system (Dong et al., 2013). Although genome-editing and shRNA technologies have not yet been tested in mosaic zebrafish cancer models, these techniques are amenable to both focused studies of single genes and large-scale genome-wide screens. When coupled with the scalability, ease of generating transgenics and the imaging capability of the zebrafish, these loss-of-function tools will likely provide an important complement to similar knockout and knock-down systems in mouse.

## Dynamic imaging of cancer cell heterogeneity

**Fig. 2.**
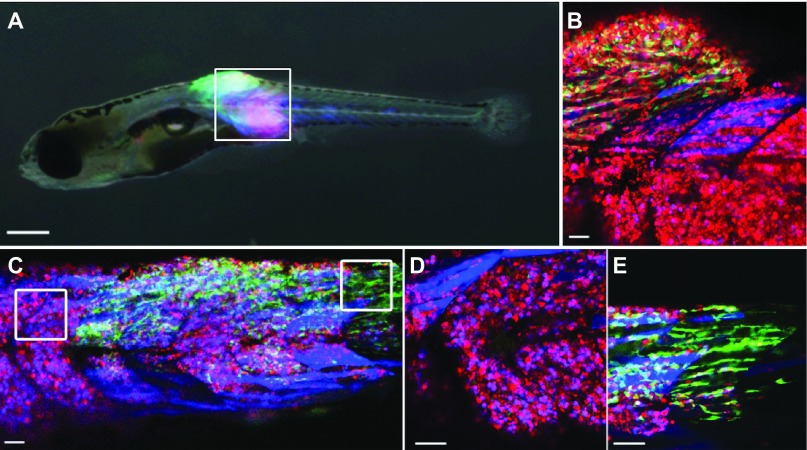
**Direct *in vivo* imaging of the functional effects of cellular heterogeneity.** (A) Whole animal imaging of a *KRAS(G12D)*-induced RMS that was created in fluorescent transgenic zebrafish. *myf5-GFP* (green) labeled the cancer stem cell fraction, *myogenein-H2-RFP* (red; appears pink in this panel) was expressed in myoblast-like cells and *mylz2-lynamCyan* (blue) was expressed in terminally differentiated tumor cells. (B) Confocal image of the boxed region shown in panel A, showing the green, red and blue labels more clearly. (C) At later stages of RMS progression, cells became compartmentalized based on differentiation status, seen as clustering of labeled cells of the same color. (D,E) Higher-magnification views of the boxed regions from panel C, showing the regional compartmentalization of RMS cells based on differentiation status. Reproduced with permission from Ignatius et al. (Ignatius et al., 2012). Scale bars: 500 μm (A); 50 μm (B–E).

In addition to genetic changes, tumor cell heterogeneity can also result from the distinct cellular states in which cancer cells are maintained. For example, numerous tumors follow a ‘cancer stem cell’ model, in which tumor-propagating cells are less differentiated than the resulting progeny that make up the bulk of the tumor mass. In these cases, differentiated cells likely contain the same genetic mutations as the self-renewing cancer stem cells, but are arrested at different stages of cellular maturation (Magee et al., 2012). Taking advantage of the well-known expression of muscle-specific genes during development, differentiation and regeneration, Ignatius and colleagues utilized muscle-specific transgenic lines to label rhabdomyosarcoma cells based on differentiation status (Ignatius et al., 2012). In this study, GFP labeled the less differentiated *myf5*-positive RMS population, whereas more-differentiated *myogenin*-and *myosin*-positive populations were labeled with mCherry and Amcyan, respectively (Ignatius et al., 2012). Labeled cell populations were easily separated by fluorescence-activated cell sorting (FACS), with subsequent microarray analysis revealing differential expression profiles between the cell types, and transplantation at varying cell doses, known as limiting-dilution transplantation, confirming that functional differences in tumor-propagating potential was based exclusively on differentiation status. *In vivo* real-time confocal imaging of primary heterogeneous RMSs showed that the *myf5*-positive cells expanded rapidly during oncogenesis. Unexpectedly, the more differentiated *myogenin*-positive cells were highly migratory, able to invade neighboring normal tissue and to intravasate. Only after the niche was established by colonizing *myogenin*-positive cells did the self-renewing *myf5*-positive cells migrate into the newly forming tumor, ultimately driving tumor expansion and progression (Fig. 2). Although this work does not differentiate between individual, genetically distinct, intratumoral clones, it highlights the power of fluorescent transgenic labeling strategies in zebrafish to provide novel insights into the functional role that cellular heterogeneity plays in cancer initiation and growth, as defined by various tumor cell states and differentiation statuses.

## Functionally assessing the role of heterogeneity in cancer progression

**Fig. 3.**
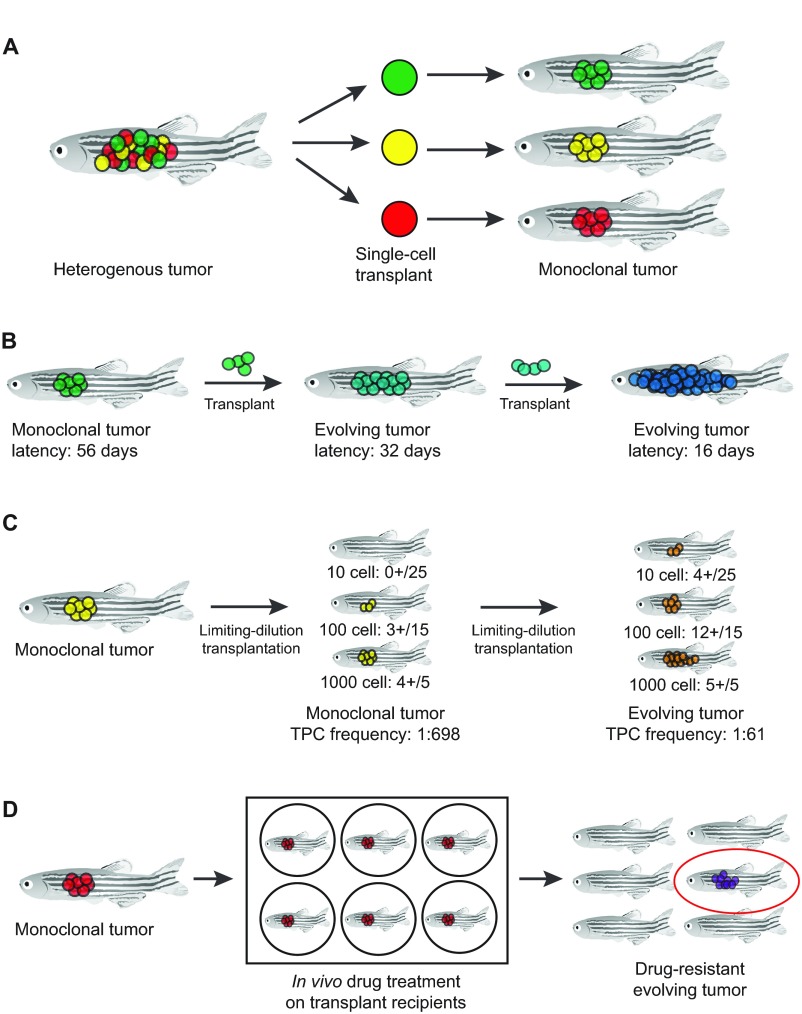
**Assessing intratumoral heterogeneity and clonal evolution using single-cell tumor transplantation.** (A) Single cells from a heterogeneous primary tumor can be isolated by FACS and transplanted by intra-peritoneal injection into genetically identical adult recipients. In this scheme, each clone within the primary tumor is represented by a different color. (A,B) Monoclonal tumors derived from engraftment of a single cell can be assessed for changes in latency (B) or tumor-propagating cell number following serial passaging (C). The frequency of tumor-propagating cells is assessed by limiting-dilution cell transplantation, in which varying doses of cells, such as 10, 100 and 1000 cells, are transplanted into recipient animals. The frequency of tumor-propagating cells is calculated based on the ratio of animals that develop a tumor compared to the total number transplanted at each dose (0+/25, 3+/15, etc.). TPC, tumor-propagating cell. (D) Transplanted zebrafish can be bathed in chemotherapies to identify subclonal variants that have become drug resistant. For each assay, monoclonal tumors and evolved clones, represented by changes in the color of the tumor, can then be harvested and examined by microarray, aCGH or other next-generation sequencing technologies to identify genes and pathways that drive these aspects of tumor progression.

The most comprehensive way to define the effects of intratumoral diversity on cancer progression is to sequence and functionally assess every cancer cell within a tumor. Although unlikely to ever be completed on such a large scale, Navin and colleagues performed CNA analysis on 100 single cells isolated from one breast tumor, identified three genetically distinct clones and reconstructed the clonal hierarchy of tumor evolution (Navin et al., 2011). Similar studies have now been completed in myeloproliferative neoplasms (Hou et al., 2012), multiple myeloma (Melchor et al., 2014) and bladder cancer (Li et al., 2012). As DNA sequencing technologies continue to advance and the associated costs decline, more of these studies will likely be completed; however, in the absence of functional annotation, it will remain a challenge to identify which mutations control specific processes, including cancer growth, progression and relapse. Although it is formally possible to functionally assess single cancer cells for differences in proliferation, metastasis and therapy resistance in mouse xenograft studies, these experiments are cost-prohibitive and leave some questions as to whether transplant of a human cell into a murine microenvironment alters the true cellular function (Quintana et al., 2008). By contrast, Smith and colleagues have previously shown that syngeneic zebrafish can be used as recipients for single-cell transplant of zebrafish *Myc*-induced T-ALL (Smith et al., 2010). In these studies, fluorescently labeled zebrafish T-ALL cells were isolated by FACS and transplanted by intra-peritoneal injection into syngeneic recipients. These experiments revealed that the absolute numbers of relapse-driving leukemia-propagating cells are much higher than previously predicted by mouse studies. In fact, one in 100 cells was capable of driving continued leukemia growth in the transplant setting (Smith et al., 2010). Building on these initial studies, we recently generated monoclonal T-ALL from a single cell using 16 different primary zebrafish T-ALL and over 1200 adult transplant recipients. The 47 T-ALL that arose were the result of engraftment of a single leukemia-propagating clone. Latency analysis, limiting-dilution cell transplantation and *in vivo* drug treatments demonstrated that clones from the same primary T-ALL were functionally heterogeneous (Blackburn et al., 2014). After serial transplant of these clones into >5000 adult syngeneic recipients, we observed that a small subset of clones continued to amass genetic and epigenetic mutations, often resulting in elevated growth rate and/or increased leukemia-propagating potential. Approximately 50% of the evolved clones activated the Akt signaling pathway, which, although not previously linked to leukemia-propagating activity, is a known prognostic indicator in T-ALL patients and has been associated with dexamethasone resistance in human T-ALL cells (Gutierrez et al., 2009; Piovan et al., 2013). Using the mosaic transgenic strategies outlined above, we went on to show that Akt activates the mTORC1 pathway to enhance leukemia-propagating cell frequency and stabilizes Myc protein to augment proliferation rates. Building on this transplantation platform, it is likely that similar single-cell transplantation studies can be completed in other zebrafish cancer models, ultimately mapping the cellular fates and functional differences within single cancer cells (Fig. 3). When coupled with genetic analysis using aCGH and whole-genome sequencing, it will be possible to identify *de novo* mutations that correlate with important features of cancer progression, likely revealing important insights into the crucial drivers of cancer heterogeneity.

## Assessing drug response in heterogeneous tumors

**Fig. 4.**
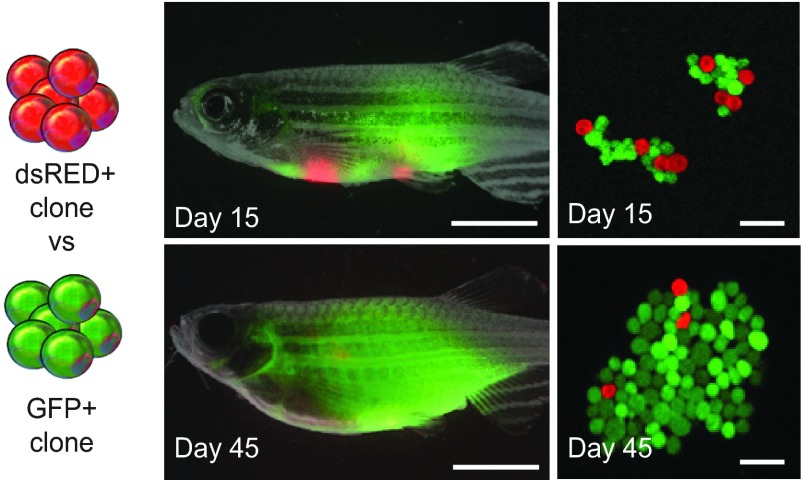
**Recapitulating heterogeneity *in vivo* through cell transplantation.** A heterogeneous T-ALL was created by mixing a single clone expressing dsRED, which had high leukemia-propagating potential and long latency, and a clone expressing GFP that had low tumor-propagating potential and short latency. Zebrafish were assessed for T-ALL growth at 15 and 45 days post-transplantation by whole-body fluorescence imaging (left) and confocal microscopy (right). Experiments revealed that proliferation and leukemia-propagating-cell frequency were regulated by different cellular processes, and that clones with high proliferation (green) outcompeted those with high leukemia-propagating frequency (red). Reproduced with permission from Blackburn et al. (Blackburn et al., 2014). Scale bars: 5 mm (left panels); 40 μm (right panels).

Zebrafish cancers are responsive to many of the same radiation and chemotherapies used to treat human cancer. Thus, it is not surprising that zebrafish have emerged as an important discovery tool for identifying novel drugs that modulate cancer growth (Yeh et al., 2009; Ridges et al., 2012; Le et al., 2013; Trede et al., 2013; Gutierrez et al., 2014). The most straightforward experiments to assess the effects of clonal heterogeneity on response to therapy will be to generate mosaic transgenic animals that bear tumors with genes of interest overexpressed or knocked out, and to screen for the emergence of therapy-resistant clones. Although large-scale drug screens are commonly completed *in vitro* using human cell lines, *in vivo* studies are probably a more accurate representation of drug response because the normal three-dimensional (3D) architecture of the tumor, pattern of heterogeneity and tumor microenvironment are maintained. In this regard, heterogeneous zebrafish tumors can be transplanted into multiple recipients, which allows for large-scale *in vivo* multi-drug screens against genotypes of interest (Chen et al., 2014). Importantly, zebrafish tumors that overexpress up to four genes simultaneously are routinely generated, suggesting that it will be possible to assess combinations of mutations for effects on therapy response (Ignatius et al., 2012). Once libraries of tumors that mimic various genetic mutations are developed, clones from genetically distinct tumors can be isolated and co-transplanted into recipient zebrafish to recapitulate intratumoral heterogeneity and animals can be examined for the effects of drug treatment on clonal outgrowth and evolution. As described above, a heterogeneous T-ALL was generated by transplanting syngeneic zebrafish with two functionally distinct T-ALL clones, ultimately demonstrating that leukemia-propagating cell frequency is not genetically coupled with proliferation rate (Blackburn et al., 2014). Because clones were engineered to express different fluorescent markers, outgrowth of the dominant clone was easily assessed by fluorescent imaging and FACS analysis (Fig. 4).

**Fig. 5.**
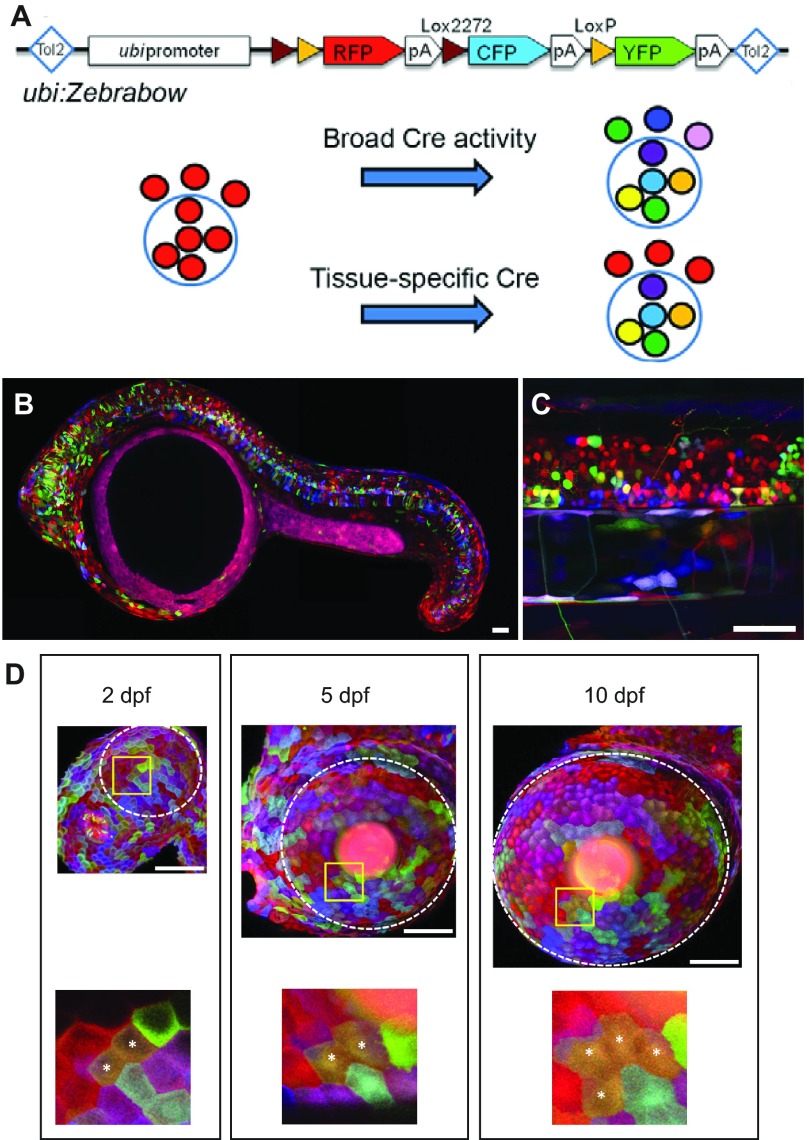
**Visualizing clonal expansion *in vivo* using Zebrabow labeling strategies.** (A, top) Schematic of the Zebrabow construct. When Cre is expressed, it can recognize the *lox2272* (brown triangles) and *loxP* (yellow triangles) sites, excising the sequence in between. Recombination only occurs between identical Lox sites because *lox2272* and *loxP* are mutually incompatible, which results in the expression of different fluorescent proteins depending on the recombination events that occur: no excision (or Cre not expressed), red; excision between *lox2272* sites, cyan; excision between *loxP* sites, yellow. Tol2, transposon; RFP, red fluorescent protein; CFP, cyan fluorescent protein; YFP, yellow fluorescent protein; pA, poly(A) site. (A, bottom) Using the construct shown, multi-cell differential fluorescent labeling can be easily achieved. The circle outlined in blue represents an organ of interest; circles outlined in black represent individual cells. Without Cre expression, all cells appear red, as shown on the left. With Cre expression in homozygotes, random combinations of yellow, blue and red are expressed by each cell, and homozygotes show increased color diversity. Cre can be expressed ubiquitously or in a tissue-specific manner using transgenic lines. (B) Embryos with Zebrabow clonal labeling at 24 hpf and (C) high-power image of the spinal cord at 3 dpf. (D) Corneal epithelial cells from in a Zebrabow animal imaged at 2, 5 and 10 dpf. A white dashed line represents the boundary of the cornea. Note the clonal expansion of cells within the cornea over this time period, which is denoted by asterisks in the enlarged boxed regions. Scale bars: 50 μm. All panels reproduced with permission from Pan et al. (Pan et al., 2013).

Although recapitulating heterogeneity through cell transplantation has its merits, an ideal system would allow for direct visualization of intratumoral heterogeneity, longitudinal analysis of clonal dominance over time, and real-time analysis of the effects of drug treatment on clone number and type. Zebrabow technology, developed from the Brainbow system used to label mouse neurons (Livet et al., 2007), has the potential to meet all of these criteria. Zebrabow is a transgenic multi-color labeling strategy in which *CRE-loxP* recombination is used to stochastically label cells with various combinations of red, cyan and yellow fluorescent proteins (Pan et al., 2013). These colors are stably maintained and inherited throughout cell division, allowing for lineage tracing and fate mapping of individual clones (Fig. 5). Co-injection of a tissue-specific *CRE–estrogen-receptor* (*ER*) and the appropriate oncogene into a Zebrabow zebrafish strain can therefore be used to generate a tumor in which different clones are individually fluorescently labeled. Using fluorescent imaging techniques, tumors can then be followed *in vivo* to assess clonal expansion and competition. Subsequent drug treatment of these animals, as well as careful genetic analysis of clones before and after drug treatment, will provide insight into how clones within a heterogeneous tumor respond to and become resistant to therapy over time.

## Concluding remarks

Clonal evolution and intratumoral heterogeneity have far-reaching clinical consequences, yet research examining this important issue is still in the early stages. Next-generation sequencing data from longitudinal patient samples will continue to be crucial, but research must also be extended into more extensive characterization of the mechanisms of clonal evolution, the identification of dominant drivers and the acquisition of drug resistance in the context of intratumoral heterogeneity. Transgenesis, transplantation assays, single-cell functional assays and direct imaging studies using zebrafish cancer models can serve as an economical, large-scale *in vivo* screening tool that can both complement and focus studies in mouse models and human cells. Understanding intratumoral heterogeneity will provide crucial biomarkers to predict cancer progression, therapy response and relapse. The ultimate hope is that identifying mutations associated with clonal heterogeneity and disease progression will lead to new personalized medicine approaches in which treatments will be tailored to the unique spectrum of mutations that define the individual clones within a patient’s tumor.
